# Defining health by addressing individual, social, and environmental determinants: New opportunities for health care and public health

**DOI:** 10.1057/jphp.2014.19

**Published:** 2014-06-19

**Authors:** Johannes Bircher, Shyama Kuruvilla

**Affiliations:** a Department of Hepatology, University of Bern, Reuelweg 20, BE, Meikirch, CH-3045, Switzerland; b The Partnership for Maternal, Newborn & Child Health, hosted by the World Health Organization, 20 Avenue Appia, Geneva, 1202, Switzerland

**Keywords:** population health, health care, determinants of health, Meikirch Model, MDGs, post-2015 development goals

## Abstract

The Millennium Development Goals (MDGs) mobilized global commitments to promote health, socioeconomic, and sustainable development. Trends indicate that the health MDGs may not be achieved by 2015, in part because of insufficient coordination across related health, socioeconomic, and environmental initiatives. Explicitly acknowledging the need for such collaboration, the Meikirch Model of Health posits that: *Health is a state of wellbeing emergent from conducive interactions between individuals' potentials, life's demands, and social and environmental determinants.* Health results throughout the life course when individuals' potentials – and social and environmental determinants – suffice to respond satisfactorily to the demands of life. Life's demands can be physiological, psychosocial, or environmental, and vary across contexts, but in every case unsatisfactory responses lead to disease. This conceptualization of the integrative nature of health could contribute to ongoing efforts to strengthen cooperation across actors and sectors to improve individual and population health – leading up to 2015 and beyond.

## Introduction

The Millennium Development Goals (MDGs) helped mobilize unprecedented global resources to promote health and socioeconomic development. Some of the MDGs, especially those related to health, may not be achieved by 2015. World leaders are now deliberating post-2015 *Sustainable Development Goals* – with *sustainable development* defined as development that meets the needs of the present without compromising the ability of future generations to meet their own needs.^1, 2^ They recognize that: “the MDGs fell short by not integrating the economic, social, and environmental aspects of sustainable development. … People were working hard – but often separately-on interlinked problems”.^2^

Promoting the health of individuals and populations is a complex endeavor – dependent upon individuals, families and communities, governments, health professionals, academics, administrators, development partners, businesses, the media, and others whose activities overlap or intertwine. A definition of health that highlights these relationships could provide a systematic way to think through required actions, and facilitate cooperation.

Our understanding of the determinants of health has broadened beyond the individual to include social determinants – by taking into account:

the unequal distribution of power, income, goods, and services, globally and nationally, the consequent unfairness in the immediate, visible circumstances of peoples lives' – their access to health care, schools, and education, their conditions of work and leisure, their homes, communities, towns, or cities – and their chances of leading a flourishing life.^3^

Environmental determinants of health, based on the definition of environmental health, include:

… all the physical, chemical, and biological factors external to a person, and all the related factors impacting behaviours … targeted towards preventing disease and creating health-supportive environments (including clean air and water, healthy workplaces, safe houses, community spaces and roads and managing climate change). This definition excludes behaviour not related to environment, as well as behaviour related to the social and cultural environment, and genetics.^4^

The far-ranging scope of social and environmental determinants of health further highlights the need for a definition of health that could link different actors and sectors.

The preamble of the World Health Organization's (WHO) constitution (1946) represents the best known definition of health – a state of “complete physical, mental and social well-being and not merely the absence of disease or infirmity”.^5^ The preamble also states that: “The enjoyment of the highest attainable standard of health is one of the fundamental rights of every human being” that “Informed opinion and active cooperation on the part of the public are of the utmost importance” and that “Governments have a responsibility for the health of their peoples which can be fulfilled only by the provision of adequate health and social measures”.

The WHO definition sets out aspirational and universal goals without much guidance on how these goals could be realized. It is not clear, for example, how governments should plan the “adequate health and social measures” to improve population health, and the requirements are likely to vary with each country's context. The translation of this definition to individuals' health also poses challenges. For example, individuals with disabilities or non-communicable and chronic conditions may subjectively feel healthy, even though by this definition they might not be considered as such. *Health*, defined as a broad goal that could mean different things to different people at different times and in different places, may hamper informed and active cooperation to achieve this goal.

In 2010, an international conference of experts presented a critique of the WHO definition of health: “It contributes to medicalization of the society, it is inadequate for chronic diseases, and it is neither operational nor measurable”. These experts recommended that a definition of health should include “the resilience or capacity to cope and maintain and restore one's integrity, equilibrium, and sense of wellbeing”.^6^ While the conference identified these useful principles, the participants stopped short of formulating a new definition of health.

Experts from a variety of disciplines have proposed alternative definitions of health, and we discuss three notable examples before explaining our own. Christopher Boorse^7^ used a statistical approach to redefine health. He proposed that statistical reference values be calculated for all possible human functions. Results that lay, for example, within the 95 per cent range would represent normal health, and results outside this range would signify disease. This definition was promoted as being quantifiable and not relying on value judgments. It was rejected – largely for being unduly disconnected from the richness and uniqueness of people's experiences of health.

Lennart Nordenfelt, working independently, proposed a normative formulation: “In order to qualify as a healthy person someone must have the ability, given standard or reasonable circumstances, to reach the person's set of vital goals”.^8, 9^ This description usefully expresses a balance between abilities and goals. Yet, when considering the needs and resources of individual patients or populations, it is difficult to establish what constitutes standard circumstances and vital goals.

In 2013, Sturmberg developed another definition concluding that health is “a personal experiential state which needs to be viewed simultaneously in terms of its somatic, psychological, social, and semiotic dimensions”.^10^ As a practicing physician interested in systems thinking he describes health as having four important features, but does not differentiate health from disease and does not analyze how health is constituted. (See Sturmberg commentary ^10^ in this special section.)

We build on our earlier publications on the nature of health^11, 12^ and extend these concepts in the Meikirch Model of Health (the Model), as explained in the section on methods. In the results section we describe the components of the Model and the dynamic interactions over time that determine individual and population health. We then discuss possible applications of the Meikirch Model of Health to strategies to improve individual health care and population health. We do not suggest that the Meikirch Model can, or should, replace existing mobilizing and operational frameworks for collective action to improve individual health care and population health. Instead, the Model could contribute to these efforts by providing a systematic way for different actors, from different sectors, to think through, develop shared understandings, and address the various determinants of health.

## Methods: Developing the Meikirch Model of Health

The Meikirch Model of Health originated in Meikirch, Switzerland – the home village of the first author (JB). After retirement from an academic career and a medical school deanship, JB started a project at the Swiss Academy of Medical Sciences about how to orient the Swiss medical care system to the challenges of the future. When the project failed to have the desired impact, a colleague suggested to JB that more far-reaching results might have been achieved from a ‘clarification of the terms' involved. Understanding the implications of this proposal, JB then started to study the term *health*. Recognizing that the many different meanings and usages of this word depended on the background and the interests of the user, he worked to tailor a new definition of health to modern needs and circumstances to facilitate cooperative action for health.

The second author (SK) approached this analysis from a global health and development perspective. Actors engaged in health and development efforts recognize that they tend to work in sectoral isolation albeit on very interlinked problems. Recognizing this challenge, the global community is currently deliberating post-2015 sustainable development goals to integrate efforts across areas of inclusive economic and social development, environmental sustainability, and peace and security.^1^ The health of individuals and populations needs to be at the heart of these collective efforts.^13^ As the Lancet Commission on Investing in Health demonstrates, healthier people can contribute more to countries' economies,^14^ and inclusive, equitable societies and sustainable environments can enhance people's health.^1^ An integrative approach is not just relevant for global development goals, but is also a fundamental principle of human rights, where rights – for example, to the highest attainable standard of health, to education, and to economic, social and cultural participation – are interdependent and indivisible.^15^ To realize human rights and development goals, there needs to be a special focus on those individuals and groups most marginalized and underserved by health and social services – often the women and children in the lowest-income communities.^13^ A shared understanding of the nature of health and its related determinants could contribute to ongoing collective efforts.

An earlier version of the Model (by JB) focused primarily on individual health care. Together we have worked to develop the Meikirch Model of Health to take into account population health considerations. We present a version here with the hope it will help many stakeholders and those collaborating across sectors to promote individuals' and populations' health.

To develop this expanded Meikirch Model of Health, we applied both deductive and inductive analysis, an approach that is set out in the *multi-grounded theory* method.^16^ The inductive phase included reviewing and codifying literature on definitions of health and critiques of these definitions. It also involved synthesizing empirical and practical experiences in clinical practice and research, with patients' experiences with health and disease, and with population health policies and programs. The authors also used deductive considerations – theories and conceptual frameworks from evolutionary biology, clinical medicine, social, anthropological, philosophical, and systems theory – to help organize and evaluate the inductive information, and to develop the Meikirch Model of Health further. Finally, we followed an interactive and iterative process with feedback from preliminary peer-reviewed publications^11, 12^ and presentations at scientific and other meetings where participants engaged in discussions of the ideas and thereby informed subsequent iterations of the Model.

## Results: Explicating the Meikirch Model of Health

The Meikirch Model of Health posits that: *Health is a state of wellbeing emergent from conducive interactions between individuals' potentials, life's demands, and social and environmental determinants.* Health results throughout the life course when individuals' potentials – and social and environmental determinants – suffice to respond satisfactorily to the demands of life. Life's demands can be physiological, psychosocial, or environmental, and vary across individual and context, but in every case unsatisfactory responses lead to disease.

**Figure 1:**
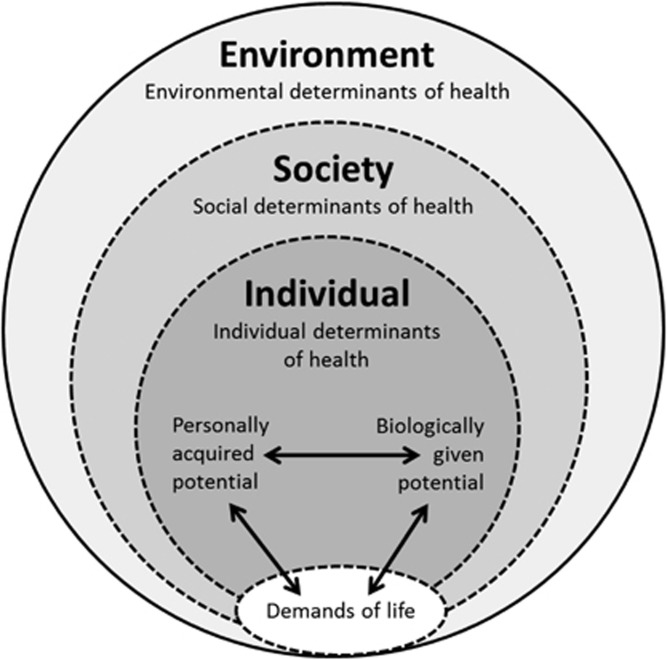
*The Meikirch Model of Health*: Health occurs when individuals use their biologically given and personally acquired potentials to manage the demands of life in a way that promotes well-being. This process continues throughout life and is embedded within related social and environmental determinants of health. Health is constituted by all three dimensions – individual, social, and environmental determinants of health.

Figure 1 depicts the Model. It comprises three main constituents of health: (i) *Individual determinants* of health that include: (a) *Demands of life* (as outlined above); and (b) *Potentials* of individuals – biologically given or personally acquired – to meet life's demands; (ii) *Social determinants* of health; and (iii) *Environmental determinants*. These determinants interact and can modify both the demands of life and potentials to respond satisfactorily to these demands. We now define and discuss each element in the Model, beginning with Individual determinants of health, followed by the Social and Environmental determinants. We then discuss how these determinants all interact as part of a complex adaptive system of health.

### Individual determinants of health

#### Demands of life

Humans are exposed to three main types of demands of life: physiological, psychosocial, and environmental demands. In the following sections we discuss how individuals use their biologically given and personally acquired potentials to process and meet these demands, and also the social and environmental factors that may facilitate or hinder this process.

- *Physiological demands*: For humans, physiological demands present themselves in many ways as functions related to input, output, and procreation. Procurement of oxygen, nutrients and water, excretion, fertilization, pregnancy and childbirth, and the maintenance of internal conditions within physiological limits (homeokinesis) are key examples. Some specific characteristics differentiate humans from other higher animals. Procreation is essential for the survival of the species, but only humans can make choices on whether, and when, to procreate. Humans deal with different conditions to meet physiological needs that vary with time and circumstance. For example, in low-income countries the main sources of food may be provided by traditional farming and, in high-income countries, by industrialized agriculture. Both food sources include external systems for storage and distribution, for instance, through local shops or supermarkets.
- *Psychosocial demands*: Psychosocial demands relate to individuals' personal development and social integration, including participation in social, economic, and political life. Personal development interlinks with social integration and is immediately apparent for newborns who need to attach to their care givers. This contributes to brain function and overall development.^17^ Each individual is exposed to various social determinants of health throughout the life course, with roles and expectations varying around the world, for example, as related to jobs, relationships, obligations to family and society, personal aspirations, and political and economic contexts. Thus, the way in which life's demands present and can be fulfilled depends very much on the specifics of the society in which an individual lives.
- *Environmental demands*: Health of individuals and populations can be affected substantially by factors in the environment, including extreme weather events, availability of clean drinking water, air pollution, food scarcity, radioactivity, and safe workplaces.^1, 4, 18^ Environmental demands of life do include protection from physical, chemical, and microbiological threats, and safe disposal of waste matter (recycling). Sustainable development focuses on environmental demands. Some of these are apparent immediately, while others could be latent for many years (for example, exposure to carcinogens from tobacco smoke or pollutants). Environmental demands are not only about protection from challenges, but also about protecting the environment to reduce environmental demands to create conditions conducive to promoting both health and sustainable development.

#### Individuals' potentials

**Figure 2:**
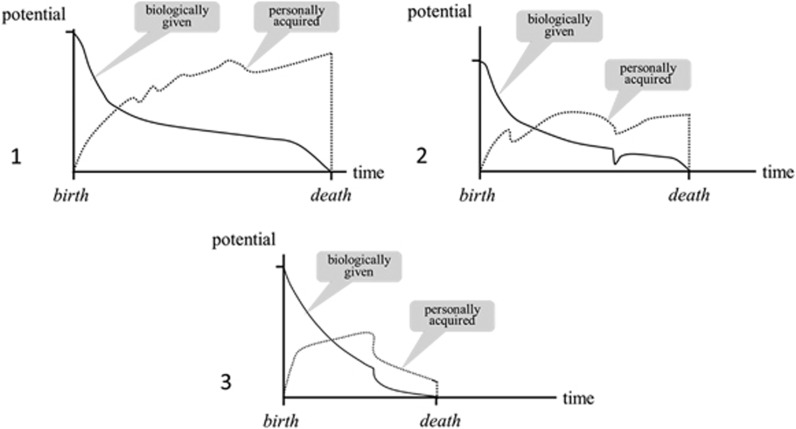
The time course of individuals' biologically given and personally acquired potentials is shown by three examples of possible time courses of the two potentials during the life of a human being. At the time of birth, biologically given potential (continuous line) has a finite value that differs from person to person, and at the time of death, it is zero. In the figure, the lines between these two points, the curves are drawn arbitrarily to illustrate these concepts. The personally acquired potential of a person (dotted lines) begins before birth, increases rapidly thereafter, and can increase throughout life, provided the individual is able to continually develop it to meet life's demands. It drops to zero at the time of death. The corresponding lines for biologically given potential in the Figure are also drawn arbitrarily for illustrative purposes. Both potentials and the demands of life are strongly influenced by social and environmental determinants as depicted in the Meikirch Model. This figure focuses on the interaction of the two potentials in the context of specific individuals. In the first example the individual has succeeded in enhancing personally acquired potential. The second may have had a crisis in puberty and later a myocardial infarction – indicated by drops in the two potentials. In the third case, both curves drop at some time due, for example, to alcoholism. At each moment in life, every individual uses her or his total potential, the composite ‘sum' of the two potentials, to try and effectively manage the demands of life.

The Model postulates that for health, each person must have the resources to meet the demands of life at any point in time. Figure 2 depicts possible interactions between individuals' biologically given and personally acquired potentials in relation to health across the life course. A common desire for a long life creates necessity to satisfy demands both in the present and for the long term. For this reason we chose the term *potential* to express both present and future resources. Individuals draw on two major potentials to process and meet life's demands: biologically given and personally acquired potentials.

- *Biologically given potential:* Our biologically given potential represents the biological basis of life. At the moment of birth it has a finite value resulting from genetic material and the quality of the pregnancy. The genetic component includes the genes themselves as well as their epigenetic regulation during pregnancy. After birth this potential diminishes throughout life, reaching zero at the time of death (Figure 2). Every somatic disease, injury, or defect diminishes the biologically given potential, either transiently or permanently.
- *Personally acquired potential:* This potential is the sum of all physiological, mental, and social resources a person acquires during life. It starts to develop in utero. As the brain and other organ systems mature, the personally acquired potential grows rapidly. For children, adolescents, and families schools and communities play a crucial part in supporting personal maturation and development of knowledge and skills. In adulthood, the development of potentials may slow down, but can increase throughout life provided an individual intends to and is able to actively promote her or his development, and lives in a health-enhancing social context. Emerging research on positive psychology highlights the importance of personally acquired potential for health. Individuals can enhance their well-being and longevity by building up positive emotions, engagement, relationships, meaning, and accomplishment.^19^ Similarly, the *salutogenesis* concept of Antonovsky proposes that individuals who understand their situation, can manage it, and find sense in it, can enhance their health.^20^

Biologically given and personally acquired potentials do not split into body and mind. Although biologically given potential is reflected in an individual's somatic constitution, many aspects of personally acquired potential also reside in the body. Individuals who have been physically active while growing up develop more athletic musculoskeletal systems than those who as youths mostly read books or played with computers. In this and many other examples, dissimilarities in personally acquired potentials are expressed as anatomical and physiological differences.

Personally acquired potential can compensate appreciably for deficiencies in biologically given potential. A person with paraplegia can become functionally independent and professionally active.^21^ By contrast, we cannot identify instances in which the biologically given potential has expanded to compensate for deficits in the personally acquired potential.

Highlighting the importance of the interaction between biologically given and personally acquired potentials for a person's well-being, the Model includes the possibility for people to consider themselves healthy despite having biomedical problems. A person might have rheumatoid arthritis and related physical impairments but if the disease is medically under control and the person has developed personal potentials to function well enough to lead a meaningful life, the individual might consider him – or herself as healthy despite having a chronic disease and related physical limitations. This holds true also in other situations where people experience common health problems. A 2007 Swiss survey^22^ found that 87 per cent of respondents reported their health as ‘good' or ‘very good'. This was despite 43 per cent reporting having had backaches, 36 per cent headaches, 35 per cent sleep disturbances, and 23 per cent other significant conditions – in the prior four weeks. Biomedical symptoms can coexist with subjective perceptions of good health.

The potentials needed to meet life's demands align with the concept of *capabilities* proposed by Amartya Sen and others.^23^ The capability approach purports that capabilities to achieve well-being are a matter of what people are able to do and to be, and thus the kind of life they are effectively able to lead. This means that promoting an individual's functional capabilities (such as the ability to participate in social, economic, and political opportunities and to make use of health care), rather than end-state utilities (health, happiness, or desire fulfillment), should be the objective of human welfare systems. It requires public or state coordination.

A difference between the capability approach and the potentials becomes evident when analyzing the fates of two people newly diagnosed with Type 1 diabetes. One living in a high-income country with adequate health care and social resources could manage the condition relatively easily – facilitated by social and environmental determinants. Another living in a low-income country − even if she or he has the same potentials as someone living in a high-income country – might not be able to afford insulin or have health care and social services required. Thus the high-income country resident may have more capabilities. In discussing personally acquired potentials the Meikirch Model of Health distinguishes between personal and social resources, whereas the capability approach combines them.

The Individual determinants of health – demands of life and people's potentials to meet them – are influenced by social and environmental determinants of health, including inequalities of resources and power and insalubrious environments, as we discuss below.

### Social determinants of health

Research shows that better social engagement, collective efficacy, and trust are associated with better health outcomes.^24^ Social factors may be positive or negative for people's well-being, including by enhancing or inhibiting the development of their potentials and by influencing the demands of life and the resources available to individuals to meet these demands. Wilkinson and Pickett identified that people's health was better in countries with less inequality in incomes.^25^ In many parts of the world poverty, living conditions, and work conditions limit the health people can achieve. The WHO Commission on Social Determinants of Health concluded:

The poor health of the poor, the social gradient in health within countries, and the marked health inequities between countries are… caused by the unequal distribution of power, income, goods, and services, globally and nationally …^2^

Michael Marmot helped define these social gradients and importantly noted that longevity is not solely related to people's income, but strongly affected by their autonomy and social participation, which are major determinants of health.^26^ He strongly emphasizes the responsibility of governments and world leaders to create circumstances that facilitate social, economic, and political participation and enable individuals and populations to improve their health.

As set out in the WHO constitution,^5^ all individuals have a right to the highest attainable standard of health, and governments have the overall responsibility to improve the health of their populations by providing adequate health and social measures. The concept of e*ntitlements* forges an essential link between legal rights and measures required to realize these rights. Sen defines entitlements as a specification of the legal rights and the resources and opportunities that enable individuals to access these rights.^27^ The 2003 health reforms in Mexico introduced a health insurance scheme known as *Seguro Popular*. Aligned with the concept of entitlements,^14^ these reforms explicitly positioned health care as a social right, and not as a commodity or a privilege. The reform arrangements included legal provisions as well as specific packages of health services.

Investments in health and social services are also important to reduce inequities, both within and across countries. The Lancet Commission on Investing in Health^13^ calls for a ‘grand convergence' within a generation. The Commission shows how investments in health could not only promote health and reduce health inequalities, but could also provide 9 to 20 times the value of the investment in social and economic benefits – as healthier people can contribute more to their societies.

Addressing the health needs of underserved and often marginalized groups, including women, children, and older people in low-income communities, is particularly important for reducing inequities and improving health.^13^ They often benefit less from health care and social services that are usually more plentiful, accessible, and of higher quality in more affluent settings. Further, in addition to communicable and non-communicable diseases that affect the whole population, they face the additional burden of morbidity and mortality related to pregnancy, and to childhood and age-related illness.

Given the linked nature of health and social and environmental determinants, governments could also consider more integrative approaches to address health, social, and environmental requirements of their populations. The example from Belo Horizonte below illustrates how this could be done.

### Environmental determinants of health

There is established evidence of important links between the environment, development, and health.^18^ These links were highlighted in1987 by the UN World Commission on Environment and Development's report – Our Common Future, ^3^ also known as the Brundtland report, that noted: “The ‘environment' is where we all live; and ‘development' is what we all do in attempting to improve our lot within that abode”.^4^

Factors in living and work environments can directly affect health. ^4, 14^ Solid fuels are an important environmental cause of disease as are waterborne contaminants. Early exposure to indoor air pollutants may damage healthy lung development, leading to a lifetime of morbidity. Adopting cleaner, more sustainable energy technologies and water sources could help promote both health and development. At the macro level, dwindling natural resources, population growth, and the effects of climate change are likely to impede improving global health.^4, 14^

A shared understanding of the nature of health, and the links between individual, social, and environmental determinants, could help promote a dialog between leaders and citizens, between public and private sectors, and with civil society and the media on the shared responsibilities to demand, provide, and use products and services in a way that is health promoting, and to put in place an appropriate and enabling environment that protects and promotes livelihood opportunities, health, and sustainable development.

## Health as a Complex Adaptive System

The Meikirch Model of Health represents health as a complex adaptive system containing ongoing interactions between individuals' potentials, the demands of life, and social and environmental determinants. This approach is in line with current thinking on complex adaptive systems.^28^ It is also aligned with the work of the philosopher John Dewey (1859–1952), who highlighted the possibility, and ethical imperative, of developing a mutually beneficial relationship among individuals as constituents of a transactive system that also comprised societies and the environment. ^29^

The Meikirch Model of Health views health as an ‘emergent property' that results from different interactions among components of a complex, adaptive system. Together the individual determinants of health, and the system as a whole – including social and environmental determinants – can develop a high degree of adaptive capacity, resulting in resilience and the ability to address ongoing and new challenges.

To achieve and maintain health over long periods, individuals must continually readjust how they use their biologically given and personally acquired potentials to respond satisfactorily to the changing demands of life – commensurate with age, gender, personal roles, culture, environment, and other factors.

Social action also is required to create circumstances that can promote individual and population health – to improve access to public goods such as education, health care, and nutritious foods, and to mitigate harm from products that cause ill health, such as tobacco and air and water pollutants; and to address inequities. This is true for low- and high-income countries.

At any point in time individuals may be subject to many demands – some immediate and some that arise from thinking about the future. Often these demands are not clearly defined. Therefore a first step is to define or diagnose the demands of life, then to prioritize which demands to respond to, and to describe and choose a satisfactory response. Such a response to life's demands might take different forms. Dewey describes three types of changes that individuals and societies (as agents) can use to resolve problematic situations:^30, 31^

- External interventions to address the agents' needs (for example, preventing diseases by building sanitation and hygiene facilities or through immunization).
- Internally oriented accommodations that agents make when circumstances cannot be changed (for example, learning to live with a chronic disease).
- Systems-wide, transformative changes in agents, environments, and the complex systems of which they are a part (for example, the evolution of species linked to changing physical environments, or deep-rooted, transformative changes in individuals and organizations in the context of socioeconomic and political reforms).

The Meikirch Model of Health postulates that if an individual's potentials and related social and environmental determinants are insufficient to respond satisfactorily to life's demands – the state is disease. When considering the balance between the potentials, determinants, and demands of life, the transition from health to disease may not be sharply demarcated. Some authors think that the two states may sometimes even overlap.^32^ Yet, in most cases the Model offers a rational, systematic approach to differentiate between the two states.

At each moment the total composite of potentials is critical for health. To meet continually changing demands of life, both (i) biologically given and (ii) personally acquired potentials are always used together. Figure 2 illustrates relative contributions of each of the two potentials over time to total potential, with advancing age favoring personally acquired potential. As we get older each of us must periodically adapt to a new relationship between our biologically given and our personally acquired potentials. Older people can continue to manage their demands of life effectively and experience well-being provided they are able to cultivate their personally acquired potential.

The usefulness of the term *potential* instead of resources becomes evident when considering a 40-year old patient with recently diagnosed arterial hypertension. Despite the disease, this person may be completely free from symptoms and feel healthy – fully able to meet the demands of life. However, the patient's future resources to meet the demands of life could be seriously jeopardized, if the high blood pressure is not treated effectively in order to avert future cerebrovascular, heart, or kidney diseases. Analogous situations would occur in considering obesity, early malignancy, Type 2 diabetes, and so on. These illuminate the need to consider potentials, not just resources at a single point in time, but through the life course.

The different determinants of health all interact and influence each other, but at different times different determinants may be the main focus of interventions. For example, general improvements in social and environmental determinants could raise living standards and promote population health overall. In individual health care, individual determinants may take precedence as a starting point for intervention. In other instances, for example in developing a public health program, all these determinants would need to be addressed.

These considerations confirm health as a state of well-being emerging from conducive exchanges among various agents as part of a complex adaptive system. Each of these components consists of many constituents, rendering their interactions even much more complex. For this reason further reductionist analytical methods to assess health may have diminishing returns, whereas complex systems approaches to understand individual and population health seem promising.^33^

## Practical Applications of the Meikirch Model

Consider, for example, the application of the Meikirch Model of Health in a clinical context with a physician using the three components of health to discuss treatment with a 27-year-old patient newly diagnosed with Type 1 diabetes mellitus. Although the treatment approach is standard, the Model offers a systematic way to think through the set of factors linked with the patient's health. This could motivate the patient.

Information for the patient: Your *demands of life* have increased because your body needs an external source of insulin throughout the day. Your *biological potential* is insufficient to meet this need. In response you must augment your *personally acquired potential* by learning the physiology of glucose and insulin and the natural history of your disease to manage it well. Management includes a special diet, physical activity, monitoring your blood glucose levels, and regularly injecting the required amounts of insulin. *Social and environmental determinants* can support you in this process. Health care providers can help monitor your health and advise you on regulating your treatment as required. You would also benefit from a range of social and environmental services, for example health insurance to pay for clinical services, including consultations and medicines. You need access to high-quality, nutritious foods. You also need environments where you can exercise and environmentally safe means to dispose of used needles and vials. Reliable sources of information on all of these issues can also support how you treat your disease. If you can manage your condition effectively, you can lead a healthy, productive and satisfying life.

While this is an oversimplification of a more complex health-care process, it serves to illustrate that the Meikirch Model could provide a framework for all participating stakeholders involved in the care of this patient, to systematically think through, organize, and demand the required resources and services to promote the patient's health. We emphasize the importance of contextual determinants in this example. It is likely patients in higher-income settings will be better able to access the required clinical, social, and environmental services to promote their health.

Next let us consider a potential application of the Meikirch Model of Health to support ongoing efforts to promote population health and sustainable development using the Belo Horizonte Food Security Program in Brazil. This Program did not explicitly use the Meikirch Model of Health, but we discuss it to highlight how a systematic approach to think through various determinants of health potentially could support similar collective efforts.

The Belo Horizonte program exemplifies the positive impacts of a truly coordinated health and sustainable development approach. Belo Horizonte is one of the most populous cities in Brazil, with 2.5 million inhabitants. In the early 1990s, about 38 per cent of its inhabitants lived below the poverty line, close to 20 per cent of children under the age of three suffered from malnutrition, and there were high rates of child mortality.

Starting in 1993, the mayor, local government, and citizens developed the Belo Horizonte Food Security policy framework. They set up a Secretariat for Food Policy and Supply, with 20 members including citizens, workers' representatives, religious and business leaders from different sectors involved with food security. These members consulted with peers and experts and advised on the design and implementation of a new system to secure widespread access to nutritious food and to raise awareness of the need for healthy eating.^34^

By 2009, evaluations in Belo Horizonte showed that 75 per cent fewer children under 5 were hospitalized for malnutrition, 60 per cent fewer children were dying, 25 per cent fewer people lived in poverty, 40 per cent of people in Belo Horizonte reported frequent intake of fruit and vegetables compared with the national average of 32 per cent.^31^ Brazil used success of the Belo Horizonte program as a model in developing its national Zero Hunger Policy. It lends credence to the value of an integrated, ethical approach to promoting health and sustainable development.

The mobilizing framework for the Belo Horizonte program was citizens' rights, and the operational framework was based on strong local governance and collective action. We are in no way suggesting that the Meikirch Model can, or should, replace existing mobilizing and operational frameworks. Instead, we propose that the Model could contribute to these ongoing efforts by offering a systematic way for different individuals and groups to think through and develop shared understandings of the determinants of health. This systematic and shared understanding could help initiate, organize, and sustain collective action.

Through the NYSASDRI Institute in India we have early feedback on better use of mother and child services and of the vaccination program, increased personal hygiene, balanced nutrition, and use of mosquito nets from explicit application of the Meikirch Model of Health in 20 tribal villages in Odisha.^35^ (See Sarangadhar Samal commentary in this special section.^35^)

## Discussion: Some Potential Applications of the Meikirch Model of Health

The Model builds on an extensive literature of theories examining and defining the nature of health, and indeed the nature of life itself.^36^ The Model is compatible with health care and public health disciplines, in that it incorporates key physiological, clinical, psychological, social, anthropological, philosophical, and systems concepts and frameworks. It specifically fulfills the postulates formulated by the group of experts reported by Huber *et al.*^6^ They wanted a definition that includes resilience, the capacity to cope and maintain and restore an individual's integrity, equilibrium, and sense of well-being. The Meikirch Model of Health satisfies these requirements. With respect to its biological and anthropological foundations, the Model may be viewed as a further development of Nordenfelt's definition that postulates a balance between abilities and goals.^8, 9^ It also encompasses Sturmberg's idea of describing health as a personal experiential state with somatic, psychological, social, and semiotic dimensions.^10^ Kuruvilla *et al* describe how “human rights principles of the interdependence and indivisibility of rights focus attention on the linkages between health, development, and human rights goals, and help promote integration of required services”.^15^ The Meikirch Model is also compatible with this approach.

One important limitation of the Meikirch Model is its theoretical and conceptual nature. Being able to assess – both quantitatively and qualitatively – individuals' potentials and the demands of life in relation to the social and environmental determinants would greatly facilitate the use of the Model in practice. The International Classification of Functioning, Disability and Health (ICF), together with the currently available tools for measuring health, disability, and quality of life, may be helpful.^37, 38^ Yet, these tools would require further development for valid evaluation of health as a complex adaptive system. Measures usefully could be developed both for individuals in terms of health status and also for population health and social and environmental determinants. Table 1 contains an indicative checklist of aspects that could be assessed. When the Model is applied to a specific situation, the analysis may reveal not one, but several or many factors that contribute to suboptimal health. If feasible, all of them need to be corrected to restore long-term health for individuals, families, or populations being considered. The procedure may also be applied to evaluate political actions.

Another limitation of the Model is that it is not yet supported by strong empirical evidence on its use or impact. In the terms set out by Dewey, the application and testing of an *Ethical Postulate*^29^ – that in a transactive system, shared responsibilities contribute to shared benefits – is ever more cogent and urgent; this is relevant in the context of individual health care and for collective action in public health and sustainable development efforts and to realize human rights.

A range of actors could, in principle, use the Meikirch Model of Health to support their work. The Model could be applied to enhance health literacy among all stakeholders involved in health care and public health,^39^ including patients and health care providers, families, and communities. Governments could use the Model to think through how best to provide adequate health and social interventions, and the related legal rights and entitlements. There also is a need for ‘systems thinking for strengthening health systems' ^40^ and to improve the coordination among all related actors. For this reason, health system planning and evaluation should include all relevant stakeholders, within and beyond the health sector, in the public and private sectors, in civil society, and in the media. It would be pertinent to conduct research on whether, and how, the Model could provide a systematic approach for a variety of stakeholders to think through their contributions to setting shared health and sustainable development goals, to support related multi-stakeholder planning and evaluation processes.

## Conclusion

We live in an interconnected world and need collective action to successfully address the challenges we face. There are several ongoing efforts aimed at building more integrative approaches to promote health and sustainable development and to realize human rights. The Meikirch Model of Health could contribute to these ongoing efforts. The Model responds to the need to develop a definition of health better suited to the operationalization and realization of the aspirations in the WHO definition, and one that facilitates systematic examination of its varied components. This could facilitate cooperation among stakeholders willing to combine forces. Health care and public health programs generally have a special need for inter-professional and inter-sectoral coordination. Using the Model, the main components – individuals' potentials, the demands of life, and the social and environmental determinants of health including the relationships among them – can be systematically identified. Such an analysis will better support operational planning than when just the broad umbrella term *health* is used. The post-2015 sustainable development agenda aims for an integrative approach across social, economic, and environmental sectors with healthy people at the heart of these efforts. Future practical experience and evaluation will reveal the extent to which the Meikirch Model of Health can contribute to this agenda and support ongoing collective action to promote individual and population health.

## Figures and Tables

**Table 1:** Using the Meikirch Model of health to support assessments of the health of individuals and populations

*CASE: (Specify and describe individual, district …)*			
*Determinants of health*	*Assessment notes*	*Plans, services, and actors required*	*Progress measures*
*Individual determinants of health*			
*Demands of life (DL)*	—	—	—
Physiological	—	—	—
Psychosocial	—	—	—
Environmental	—	—	—
*Individual potentials*	—	—	—
Biologically given potential (BP)	—	—	—
Personally acquired potential (PP)	—	—	—
*Social determinants of health*			
Social determinants of health (SD)	—	—	—
*Environmental determinants of health*			
Environmental determinants of health (ED)	—	—	—
*Key interactions (examples)*			
DL to BP and PP	—	—	—
SD to DL, BP and PP	—	—	—
ED to DL, BP and PP	—	—	—
*Complex adaptive systems*			
Systems responses in relation to different situations, for example, causal loop analyses	—	—	—
*Health outcomes*			
Based on the specific application/s for individual and population health	—	—	—

Specify the case for assessment, for example, an individual, a district, and so on. In each case the three main constituents of health and the key interactions among these components could be investigated, including at a systems level. Recognizing that more detailed, standardized assessments and tests might be required in each section, and that not all these assessments may be required in all cases, this table provides an overview of a possible checklist or worksheet to systematically think through the individual, social, and environmental determinants of health using the Meikirch Model.

## References

[bib1] United Nations World Commission on Environment and Development 1987 Our Common Future Oxford: Oxford University Press

[bib2] United Nations, High-Level Panel of Eminent Persons on the Post-2015 Development Agenda 2013A new global partnership: Eradicate poverty and transform economies through sustainable development, , http://www.post2015hlp.org , accessed 14 January 2014.

[bib3] Commission on Social Determinants of Health 2008CSDH Final Report: Closing the Gap in a Generation: Health Equity through action on the Social Determinants of Health. World Health Organization, Geneva, , http://www.who.int/social_determinants/thecommission/finalreport/en/ , accessed 19 February 2014. 19385432

[bib4] World Health Organization 2014Health topics: Environmental health, , http://www.who.int/topics/environmental_health/en/ .

[bib5] Preamble to the Constitution of the World Health Organization as adopted by the International Health Conference, New York, 19–22 June 1946; signed on 22 July 1946 by the representatives of 61 States (Official Records of the World Health Organization, no. 2, p. 100) and entered into force on 7 April 1948.

[bib6] Huber M. 2011 How should we define health British Medical Journal 26 (343 d4163 21791490 10.1136/bmj.d4163

[bib7] Boorse C. 1997 A rebuttal on healthIn: J.M. Humber and R.F. Almeder (eds.)What is Disease? Totowa, NJ: Humana Press 1 134

[bib8] Nordenfelt L. 1995 On the Nature of Health Dordrecht, Boston, London: Kluwer Academic Pressp.212

[bib9] Nordenfelt L. 2007 The concepts of health and illness revisited Medicine, Healthcare and Philosophy 10 (1 5 10 10.1007/s11019-006-9017-3 16955344

[bib10] Sturmberg J.P. 2013 Health: A personal complex adaptive stateIn: J.P. Sturmberg and C.M. Martin (eds.)Handbook of Systems and Complexity in Health New York, Heidelberg, Dordrecht, London: Springer Science+Business Media 231 242doi:10.1007/978-1-4614-4998-0_15

[bib11] Bircher J. 2005 Towards a dynamic definition of health and disease Medicine, Healthcare and Philosophy 8 (3 335 341doi:10.1007/s11019-005-0538-y 16283496

[bib12] Bircher J. Wehkamp K.H. 2011 Health care needs need to be focused on health Health 3 (6 378 382doi:10.4236/health.2011.36064

[bib13] Presern C. 2013 Post-2015 working group of the partnership for maternal, newborn & child health placing populations' health at the heart of the post-2015 agenda Bulletin of the World Health Organization 91 (7 467 467 23825869 10.2471/BLT.13.125146 PMC3699804

[bib14] Jamison D. 2013 Global health 2035: A world converging within a generation The Lancet 382 (9908 1898 1955 10.1016/S0140-6736(13)62105-4 24309475

[bib15] Kuruvilla S. 2012 The millennium development goals and human rights: Realizing shared commitments Human Rights Quarterly 34 (1 141 177

[bib16] Goldkuhl G. Cronholm S. 2010 Adding theoretical grounding to grounded theory: Toward multi-grounded theory International Journal of Qualitative Methods 9 (2 187 205

[bib17] Sullivan R. Sloan A. Kleinhaus K. Burtchen N. 2011 Infant bonding and attachment to the caregiver: Insights from basic and clinical science Clinics in Perinatology 38 (4 643 655 22107895 10.1016/j.clp.2011.08.011 PMC3223373

[bib18] Haines A. Alleyne G. Kickbusch I. Dora C. 2012 From the earth summit to Rio+20: Integration of health and sustainable development Lancet 379 (9832 2189 2197 22682465 10.1016/S0140-6736(12)60779-X

[bib19] Seligman M.E.P. 2011 Flourish: A Visionary New Understanding of Happiness and Wellbeing New York: Free Press 182 220

[bib20] Antonovsky A. 1987 Unravelling the Mystery of Health – How People Manage Stress and Stay Well San Francisco, CA: Josses-Bass Publishers

[bib21] Peter C. Müller R. Cieza A. Geyh S. 2012 Psychological resources in spinal cord injury: A systematic literature review Spinal Cord 50 (3 188 201 22124343 10.1038/sc.2011.125

[bib22] Lieberherr R. Marquis J.F. Storni M. Wiedenmayer G. 2007 Gesundheit und Gesundheitsverhalten in der Schweiz [Health and Health Behavior in Switzerland] Neuchâtel: Bundesamt für Statistik

[bib23] Stanford Encyclopedia of Philosophy 2011The capability approach, , http://plato.stanford.edu/entries/capability-approach/ , accessed 10 January 2014.

[bib24] Kawachi I. 2001 Social capital for health and human development Development 44 (1 31 35

[bib25] Wilkinson R. Pickett K. 2009 The Spirit Level: Why Equality is Better for Everyone London: Penguin Books

[bib26] Marmot M. Allen J. Bell R. Bloomer E. Goldblatt P. 2012 Consortium for the European review of social determinants of health and the health divide Lancet 380 (9846 1011 1129 22964159 10.1016/S0140-6736(12)61228-8

[bib27] Sen A. 1982 The right not to be hungryIn: G. Floistad (ed.) Contemporary Philosophy, 2 the Hague, the Netherlands: Martinus Nijhoff

[bib28] Allen P. Magure S. McKelvey B. 2011 The Sage Handbook of Complexity and Management Sage Publications

[bib29] Dewey J. 1891/1999 Outlines of a critical theory of ethicsIn: J.A. Boydston and L.A. Hickman (eds.)The Collected Works of John Dewey, 1882–1953. The Electronic Edition Carbondale and Edwardsville; Charlottesville: Southern Illinois University Press; InteLex ‘Past Masters' series

[bib30] Dewey J. 1934 A Common Faith New Haven, CT: Yale University Press

[bib31] Joas H. 1996 The Creativity of Action Chicago, IL: The University of Chicago PressOriginally published by Suhrkamp Verlag 1992.

[bib32] Law I. Widdows H. 2008 Conceptualizing health: Insights from the capability approach Health Care Anal 16 (4 303 314doi:10.1007/s10728-007-0070-8 17922192

[bib33] Began J.W. Zimmerman B. Dooley K. 2003 Health Care Organization as Complex Adaptive SystemsIn: S.M. Mick and M. Wyttenbach (eds.)Advances in Health Care Organization Theory San Francisco, CA: Jossey-Bass 253 288

[bib34] World Future Council 2009Celebrating the Belo Horizonte food security programme, Future Policy Award 2009: Solutions for the food crisis, , http://www.worldfuturecouncil.org/fileadmin/ user_upload/PDF/Future_Policy_Award_brochure.pdf , accessed 21 February 2014.

[bib35] Samal S. Bircher J. 2013What is Health? Why Do We Need to Know it? (Manual for implementing the Meikirch Model to improve health care), NYSASDRI Company, Bhubaneswar, Odisha, India, , http://www.nysasdri.org/pdf/Meikirch_Model/Meikirch_Model_ 2nd_edition.pdf , accessed 14 January 2014.

[bib36] Maklem P.T. Seely A. 2010 Towards a definition of life Perspectives in Biology and Medicine 53 (3 330 340 20639603 10.1353/pbm.0.0167

[bib37] World Health Organization 2008 International Classification of Functioning, Disability and Health Geneva, Switzerland: World Health Organization 10.1097/MRR.0b013e3282f4525c 18277203

[bib38] Üstün T.B. Kostanjsek N. Chatterji S. Rehm J. 2010Measuring health and disability Manual for WHO Disability Assessment Schedule (WHODAS 2.0). World Health Organization, Geneva, Switzerland, , http://whqlibdoc.who.int/publications/2010/9789241547598_eng.pdf , accessed 19 February 2014.

[bib39] Pleasant A. Kuruvilla S. 2008 A tale of two health literacies: Public health and clinical approaches to health literacy Health Promotion International 23 (2 152 159 18223203 10.1093/heapro/dan001

[bib40] Alliance for Health Policy and Systems Research, World Health Organization 2009 Systems Thinking for Health Systems Strengthening Geneva, Switzerland: World Health Organization

